# Supplementary material for: Optimized use of the FilmArray Meningitis/Encephalitis panel for early discontinuation of antibiotic therapy
Source: Microbiol Spectr. 2026 May 6;14(6):e00566-26. doi: 10.1128/spectrum.00566-26 (PMC13227975; doi:10.1128/spectrum.00566-26)
Supplement: Supplemental material — Fig. S1–S4; Tables S1–S4. [file spectrum.00566-26-s0001.docx]

Online Supplement to:

**Optimized Use of the FilmArray® Meningitis/Encephalitis Panel for Early Discontinuation of Antibiotic Therapy**

Nathan Nicolau-Guillaumet^1^, Marin Moutel^2^, Chloé Plouzeau Jayle^3^, Gauthier Pean de Ponfilly^4^, Virginie Courbin^5^, Anne-Gaëlle Ranc^6^, Hélène Revillet^7^, Bruno Mourvillier^8^, Maxime Hentzien^2^, Anaëlle Muggeo^1^, Thomas Guillard^1,*^, on behalf of the GMC Study Group.

Correspondence to: [tguillard@chu-reims.fr](mailto:tguillard@chu-reims.fr)

This PDF file includes:

Figures. S1 to S4

Tables S1 to S4

**Material and Methods**

**Data harmonization**

In some centers, the lower reporting limit for the CSF white blood cell (WBC) count was below 2/mm³; therefore, all cell counts at or below 2/mm³ were adjusted to 2/mm³. Similarly, one center did not report CSF red blood cell counts below 100/mm³, so all reports with 100 or fewer red blood cells per mm³ were standardized to 100/mm³. Finally, in the cases where the direct examination of the CSF was not reported, the result was modified to “negative,” as the clinical impact was equivalent.


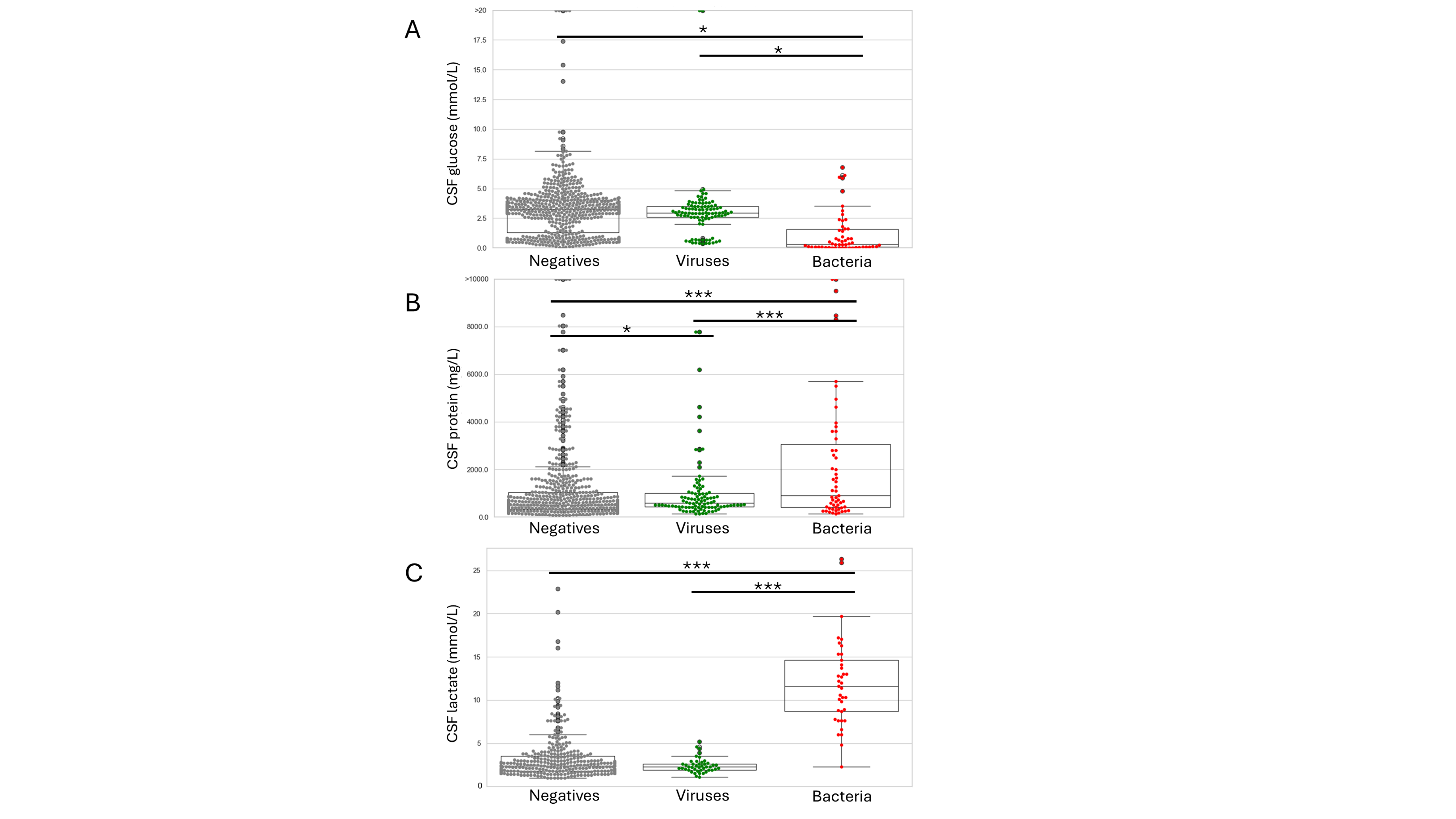


**Figure S1. CSF biochemistry.** (A) Glucose. (B) Proteins. (C) Lactates. Statistical Analyses: Kruskal-Wallis Test complemented by Mann-Whitney U Test. **p<0.5* ***p<0.001* ****p<0.0001*.


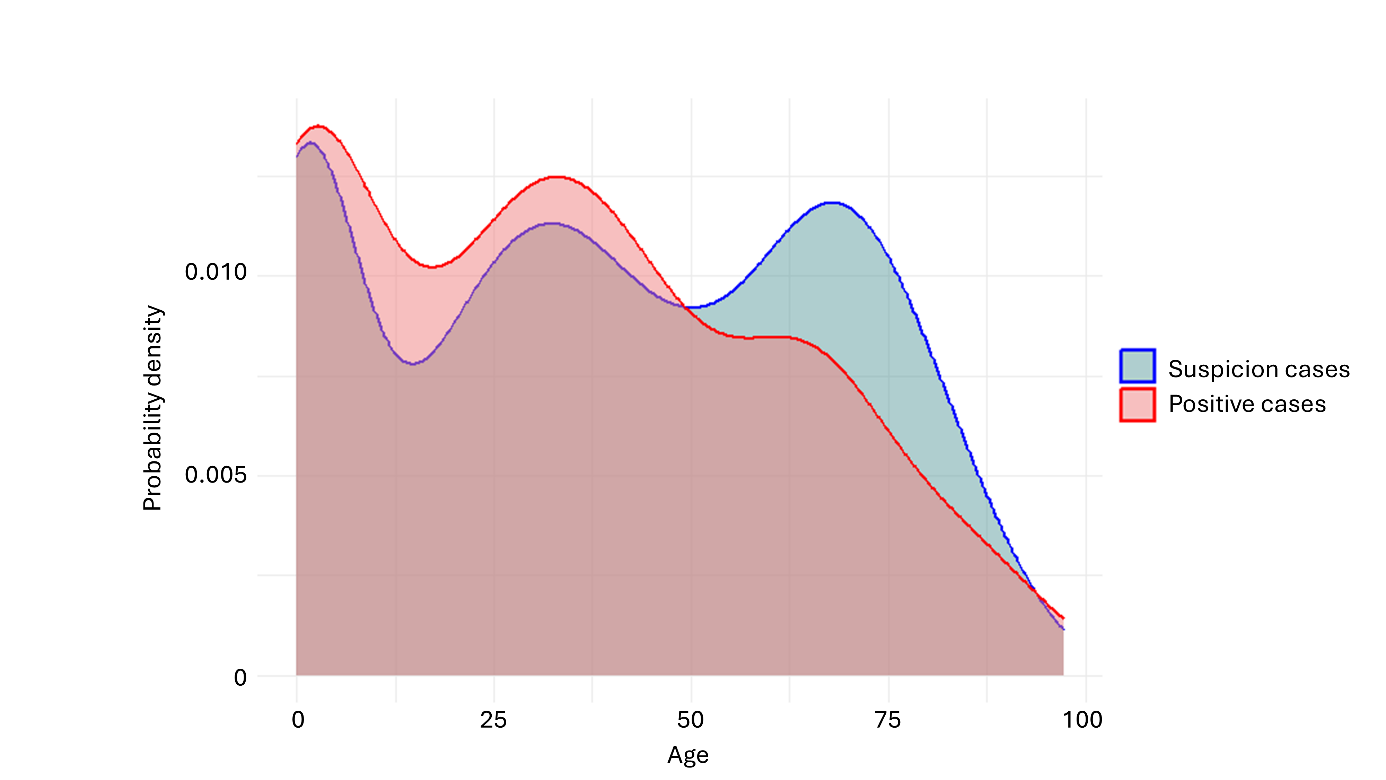


**Figure S2. Probability density of suspicion and positive cases of meningitis/encephalitis according to age.**


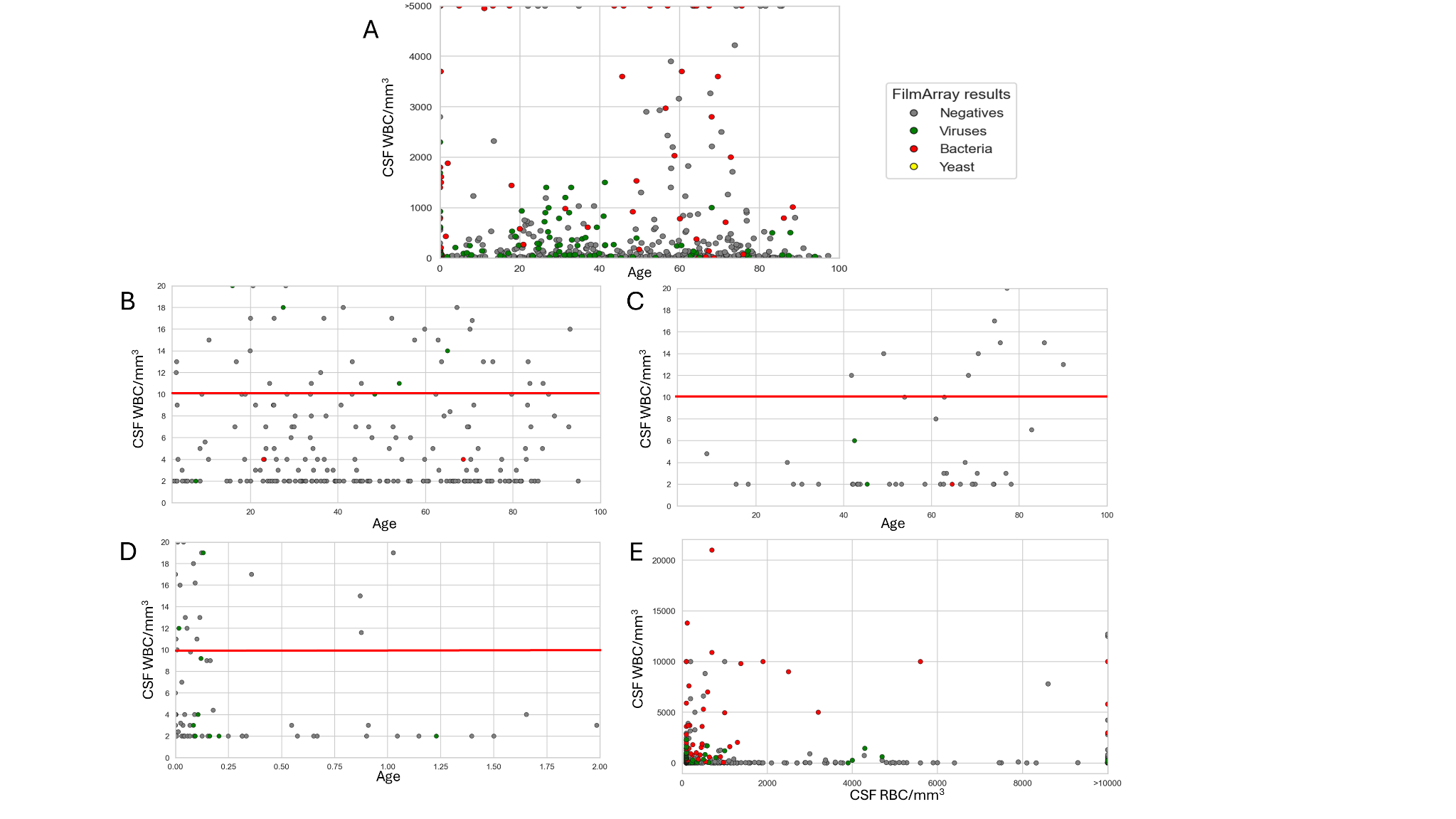


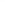

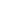


**Figure S3. Distribution of CSF cytology and FAME results.** (A) According to age: All patients. (B) Non immunocompromised patients over the age of 2 with CSF cytology between 0 and 20 WBC/mm^3^. (C) Immunocompromised patients over the age of 2 with CSF cytology between 0 and 20 WBC/mm^3^. (D) Patients immunocompromised and not immunocompromised under the age of 2 with CSF cytology between 0 and 20 WBC/mm^3^. (E) According to RBC/mm^3^.

**
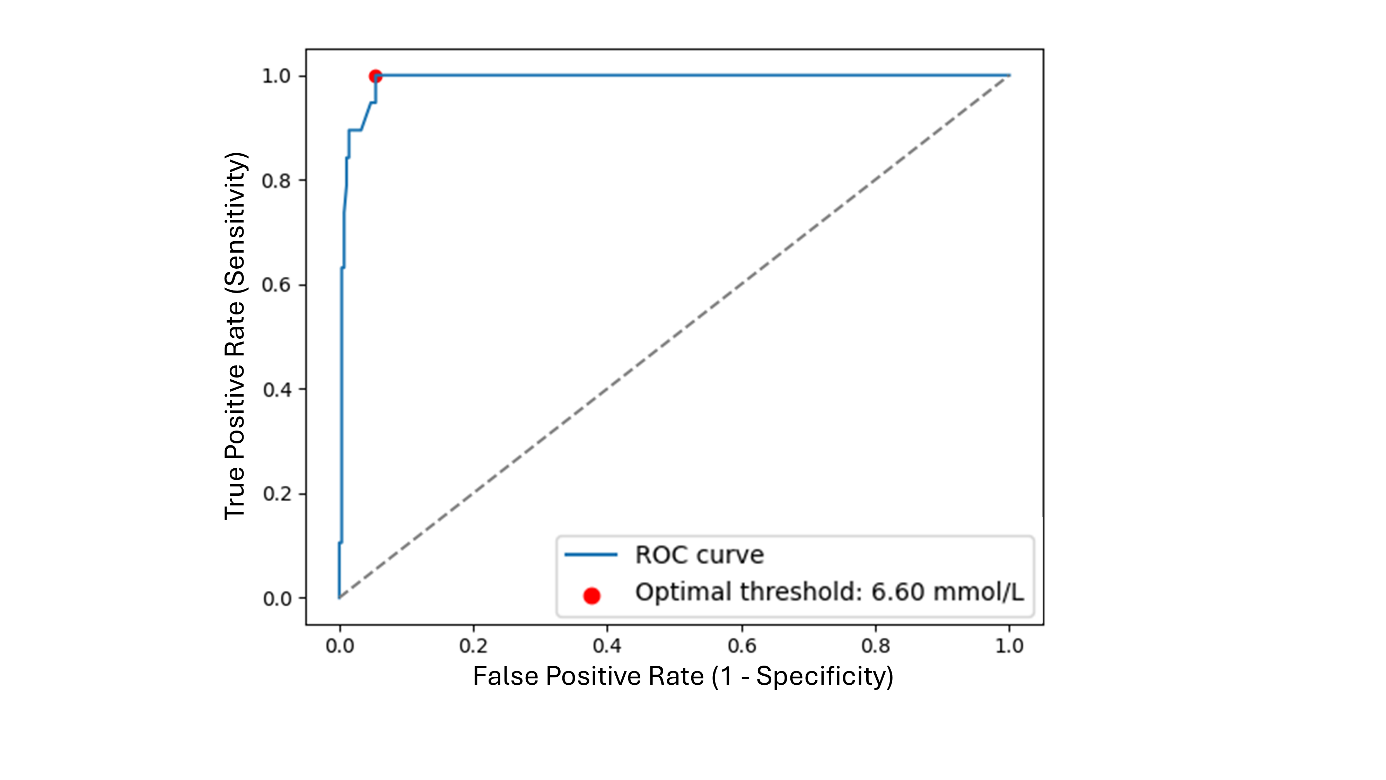
**

**Figure S4. CSF lactates ROC curve.**

**Table S1. Immunodepression criteria**

| Criteria | - Patients with asplenia or hyposplenia (including major sickle cell disease) - Patients with hereditary immunodeficiencies - Patients with uncontrolled HIV infection or CD4 count <500/mm³ - Patients undergoing chemotherapy - Patients with metastatic solid tumors or hematologic malignancies - Patients who have undergone solid organ transplantation - Patients who have received hematopoietic stem cell transplantation - Patients treated with immunosuppressants, biotherapy, and/or corticosteroids for autoimmune or chronic inflammatory diseases - Patients treated with corticosteroids ≥10 mg/day for ≥30 days or >5 mg/kg for >5 days - Patients with nephrotic syndrome - Patients with decompensated cirrhosis |
| --- | --- |

**Table S2. Number of patients included by each center.**

| **Center** | **Number of patients included n(%)** |
| --- | --- |
| Lyon | 98 (12%) |
| Paris Bégin | 84 (11%) |
| Paris St Joseph | **1**67 (21%) |
| Potiers | 186 (24%) |
| Reims | 170 (22%) |
| Toulouse | 81 (10%) |

**Table S3. Details of cases <10 WBC/mm^3^**

|  | **<10 WBC/mm^3^** | **Immunocompromised** | **<2 years old** | **Contamination** |
| --- | --- | --- | --- | --- |
| **Bacteria** |  |  |  |  |
| *Haemophilus influenzae* | 2 | 0 | 0 | 2 |
| *Listeria monocytogenes* | 1 | 1 | 0 | 0 |
|  |  |  |  |  |
| **Viruses** |  |  |  |  |
| Enterovirus | 6 | 0 | 5 | 0 |
| HHV-6 | 1 | 0 | 1 | 0 |
| Parechovirus | 1 | 0 | 1 | 0 |
| VZV | 1 | 1 | 0 | 0 |
| CMV | 1 | 1 | 0 | 0 |

**Table S4. Details of identified infectious agents.**

|  | **Positive-FAME** | **Negative-FAME** | **Positive-culture** | **Community-acquired** | **Contamination** |
| --- | --- | --- | --- | --- | --- |
| **In-panel bacteria** |  |  |  |  |  |
| *Escherichia coli* K1 | 4 | 0 | 1 | 4 | 0 |
| *Heamophilus influenzae* | 10 | 1 | 7 | 10 | 2 |
| *Listeria monocytogenes* | 8 | 0 | 6 | 8 | 0 |
| *Neisseria meningitidis* | 9 | 0 | 3 | 9 | 0 |
| *Streptococcus agalactiae* | 9 | 0 | 5 | 9 | 0 |
| *Streptococcus pneumoniae* | 15 | 0 | 7 | 15 | 0 |
| **Off-panel bacteria** |  |  |  |  |  |
| *Enterbacter cloacae* |  | 1 | 1 | 0 | 0 |
| *Escherichia coli* non K1 |  | 1 | 1 | 1 | 0 |
| *Staphylococcus aureus* |  | 1 | 1 | 1 | 1 |
| *Staphylococcus capitis* |  | 1 | 1 | 1 | 1 |
| *Staphylococcus epidermidis** |  | 3 | 3 | 1 | 3 |
| *Streptococcus equi* |  | 1 | 1 | 0 | 0 |
| *Streptococcus gallolyticus* |  | 1 | 1 | 0 | 0 |
| *Streptococcus oralis* |  | 1 | 1 | 1 | 0 |
| *Streptococcus salivarius* |  | 1 | 1 | 1 | 1 |
| **In-panel virus** |  |  |  |  |  |
| Cytomegalovirus | 2 | 0 |  | 2 | 0 |
| Enterovirus | 56 | 0 |  | 56 | 0 |
| Herpes simplex virus 1 | 9 | 1 |  | 9 | 0 |
| Herpes simplex virus 2 | 12 | 0 |  | 12 | 0 |
| Human herpesvirus 6 | 4 | 0 |  | 4 | UD |
| Human parechovirus | 3 | 0 |  | 3 | UD |
| Varicella zoster virus | 14 | 0 |  | 14 | 0 |
| **In-panel yeast** |  |  |  |  |  |
| *Cryptococcus neoformans/gattii* | 1 | 0 | 1 | 1 | 0 |
| **Off-panel yeast** |  |  |  |  |  |
| *Candida parapsilosis** |  | 1 | 1 | 1 | 1 |

*One case of culture with two pathogens. UD: Unavailable data.
